# Improved Socio-Emotional and Behavioral Functioning in Students with Autism Following School-Based Smartglasses Intervention: Multi-Stage Feasibility and Controlled Efficacy Study

**DOI:** 10.3390/bs8100085

**Published:** 2018-09-20

**Authors:** Arshya Vahabzadeh, Neha U. Keshav, Rafiq Abdus-Sabur, Krystal Huey, Runpeng Liu, Ned T. Sahin

**Affiliations:** 1 Brain Power, 1 Broadway, Cambridge, MA 02142, USA; arshya@brain-power.com (A.V.); neha@brain-power.com (N.U.K.); rafiq@brain-power.com (R.A.-S.); huey.k@husky.neu.edu (K.H.); runpeng@brain-power.com (R.L.); 2 Department of Psychiatry, Massachusetts General Hospital, Boston, MA 02114, USA; 3 Department of Electrical Engineering and Computer Science, Massachusetts Institute of Technology, Cambridge, MA 02142, USA; 4 Department of Psychology, Harvard University, Cambridge, MA 02138, USA

**Keywords:** autism, technology, augmented reality, digital health, digital medicine, edutech, data, artificial intelligence, special education

## Abstract

**Background:** Students with Autism Spectrum Disorder (ASD) commonly demonstrate prominent social communication deficits, symptoms of attention-deficit/hyperactivity disorder, and chronic irritability. These challenges hinder academic progress and frequently persist despite educational, behavioral, and medical interventions. An assistive smartglasses technology may aid these individuals, especially if the technology is efficacious in ecologically-valid school settings. This study explored the feasibility and efficacy of Empowered Brain, a computerized smartglasses intervention designed as a socio-emotional behavioral aid for students with ASD. **Methods:** This two-part six-week study involved four school children with ASD from a public elementary school. The study incorporated an initial three-week feasibility stage followed by a three-week controlled longitudinal efficacy stage. Both stages involved the use of a twice-daily socio-emotional intervention with the smartglasses. Educators completed pre-intervention and post-intervention Aberrant Behavioral Checklist (ABC) ratings at the start of the feasibility stage, and weekly during the efficacy stage. Primary outcome measures were improvements in the ABC subscales of irritability, hyperactivity, and social withdrawal. **Results:** Students in both feasibility and efficacy stages demonstrated improvements (decreases) in irritability, hyperactivity, and social withdrawal compared to a baseline period and control periods, respectively. Participants in the controlled efficacy stage demonstrated decreased ABC subscale scores of 90% for irritability, 41.6% for hyperactivity, and 45.6% for social withdrawal. An intervention exposure-response improvement in irritability and hyperactivity was found during the efficacy stage. Educators rated the technology as superior or vastly superior compared to other assistive technologies. **Conclusion:** A substantial number of school children with ASD demonstrate chronic and impairing cognitive and behavioral challenges. This study provides evidence that Empowered Brain, a smartglasses-based socio-emotional aid for autism, is both feasible and efficacious in improving symptoms of social withdrawal, irritability, and hyperactivity in students with autism. The improvement is demonstrated as part of a longitudinal school-based intervention. Further studies involving larger samples and incorporation of randomized controlled trial methodology are underway to further elucidate the impact of this technology.

## 1. Introduction

Autism Spectrum Disorder (ASD) is a childhood onset neurodevelopmental disorder that is principally characterized by impairment in social communication, and the presence of a restricted/repetitive range of interests [1]. While decreased reciprocal social interaction is frequently seen among children with ASD, these individuals also commonly demonstrate irritability [2] and symptoms of attention-deficit/hyperactivity disorder (ADHD) [3], such as hyperactivity, inattention, and impulsivity. These symptoms not only burden individuals with ASD and their families, but they may jeopardize therapeutic and educational efforts [4,5,6].

Current and historic descriptions of ASD have universally recognized the markedly impaired social communication seen in this condition [1,7,8,9]. Impairment in social communication is the most prominent and central deficit in ASD. It is, therefore, invariably true that most children with ASD will display altered patterns of social interaction with their peers and adults. Childhood reciprocal social engagement may be the most significant predictor of adult social outcomes [10], an important consideration given the poor social functioning of adults with ASD [11]. Little progress has been made on developing a pharmacological intervention to improve these social communication challenges. However, there are various attempts at using behavioral therapies to improve social skills. Evidence suggests that social interactions can be taught and that individuals can learn to demonstrate these interactions in typical settings, and gradually accrue the benefits of such approaches [12]. A range of psychosocial interventions have shown promise in improving social communication and other developmental markers, although evidence for the longer term impact of such approaches remains limited [13].

In addition to social communication deficits, up to 88% of children with ASD display marked irritability [14,15]. Irritability may manifest as aggression, tantrums, problematic behavior, and self-injury. It is the principal factor that leads caregivers to seek treatment for a person with ASD [16], and has been identified as the key predictor of caregiver stress [17]. Irritability in ASD has also been linked to increased risk of depression and anxiety, and impaired home and school functioning [18]. The underlying cause of irritability in ASD has not been fully elucidated, but aberrant emotional regulation may play a central role [19,20]. There are several treatment options to help address irritability, which often focus on ruling out an underlying medical cause of irritability, improving functional communication, and addressing psychosocial stressors (4). Cognitive behavioral therapy shows promise in improving emotional regulation in ASD, potentially decreasing irritability through these means [19,21]. Additionally, in the most severe cases, consideration of an antipsychotic may be warranted, typically aripiprazole [22] or risperidone [23]. Both of these medications have proven to be efficacious in reducing severe irritability that is associated with ASD [22,23], but often result in problematic side effects including sedation, weight gain, metabolic dysfunction, and altered prolactin levels [24,25].

Caregivers of children with ASD have also demonstrated a variety of strategies to address irritability that aim to decrease distress, provide structure, and improve children’s compliance with everyday activities [5]. These techniques include verbal reminders [26], physical prompts [27], linking activities to a child’s unique interests [28], providing choices [29], using a reward system [30], employing distracting activities [31], and providing positive praise for a child’s behavior. Parents note that technology, including smartphones and tablets, plays a key role in addressing these behavioral challenges [5,30]. Irritability and problem behaviors may manifest as a result of difficulties in communication [32,33], and may be reduced when more effective communication is achieved [34]. While many of these parental approaches could be provided through digital means, there are currently no augmented reality interventions described in the literature that target irritability and/or aggression in people with ASD.

While social communication deficits and irritability are common in ASD, up to a third of individuals with ASD may also be diagnosed with ADHD [35], a neurodevelopmental condition that manifests as functionally impairing levels of hyperactivity, inattention, and impulsivity [1]. Children with ADHD, and those who demonstrate ADHD-symptoms without a formal diagnosis, have worse academic and educational outcomes compared to their non-ADHD peers [36]. ADHD is associated with lower educational achievement in reading, writing, and math [37], accompanied by lower rates of graduation, and higher rates of detention and expulsion [36]. The combination of ASD and ADHD appears to be particularly impairing, and results in worse outcomes, including poorer quality of life [38] and lower levels of adaptive functioning [39], compared to ADHD alone. Additionally, these dual-diagnosis individuals have higher levels of anxiety and reduced working memory performance [40]. The presence of symptoms of ADHD in children with ASD are associated with impaired academic, social, and emotional performance compared to children with ASD alone [41]. Individuals with ASD and ADHD appear to have a lower likelihood of receiving ADHD treatment [42], and respond less favorably and experience more side effects to stimulant medication, the mainstay of pharmacological ADHD treatment [43,44].

## 2. Addressing Social Communication, Irritability, and ADHD Symptoms through Technology

A range of innovative technologies has been demonstrated to be an effective intervention in addressing the needs of people with ASD [45], including helping to improve challenging behavior, social communication, and academic performance [46]. New digital tools may help to more efficiently monitor and assess an individual’s behavior and cognitive processes compared to more traditional human-based assessments [47]. Augmented reality is one such technology, and has been studied as a tool to improve the educational experience of children with special needs, including children with ASD and ADHD [48]. Specifically, augmented reality interventions have been shown to have utility to improve ASD-related socio-emotional functioning [49,50,51,52] and attention [53]. Yet, despite these reports, the rapidly advancing availability and functionality of new technologies necessitates a corresponding vigor in researching their potential utility and safety as an intervention for people with ASD.

We are also witnessing the combination of previously separate innovative technologies in order to enhance their functionality and efficiency. These technological convergences are made possible through advances in material science, computer science, and engineering. One such example is the use of sensor rich computerized smartglasses that are able to utilize both augmented reality and artificial intelligence to provide for personalized socio-emotional coaching. We have previously described one such technology, referred to in this study as the Empowered Brain, and demonstrated preliminary evidence of its feasibility [54], usability [55], desirability [56], and safety in ASD populations [57]. Additionally, pilot data found the technology to be associated with improvement in social communication, decreased irritability and ADHD symptoms in people with ASD [58]. Early school-based studies have shown some early evidence of feasibility, usability, and efficacy [59,60]. Various other groups are also developing unique Google Glass-based technology to aid social communication skills in ASD. It appears that the type of software and interface used in such technology has considerable impact on usability and feasibility. Some researchers have found similarly high rates of usability and feasibility as our previous work, with all of their consented participants completing their study [61], while others have faced considerable challenges with over 40% subject dropout due to withdrawal of parental consent, inability to incorporate use of the technology to real-world schedules, or a lack of compliance with the proposed usage plan [62].

In this study we sought to investigate the socio-emotional and behavioral effects of a longitudinal intervention program using the Empowered Brain on students with ASD. We aimed to:

1. Assess the feasibility of the longitudinal use of this technology in real-world classrooms.
2. Assess the efficacy of the intervention by measuring irritability, hyperactivity, and social withdrawal, using a validated scale.
3. Allow facilitation of the intervention by a range of educators that typically provides behavioral interventions in the classroom, including special education teachers and speech and language therapists (SLTs).
4. Provide the intervention longitudinally over two weeks with twice-daily interventions during school days, overcoming prior limitations focusing on outcomes related to a single intervention session.
5. Have multiple raters: in this study, every student had pre- and post-intervention ratings completed by a special educator teacher and an SLT.

We used the Aberrant Behavioral Checklist (ABC) [63], a widely-used validated scale for measuring the effects of treatment in people with developmental disabilities [64]. Our primary outcome measures were changes in irritability, hyperactivity, and social withdrawal, as determined by the corresponding ABC subscales. The ABC consists of five different subscales (Irritability, Agitation, Crying; Lethargy, Social Withdrawal; Stereotypic Behavior; Hyperactivity, Noncompliance; and Inappropriate Speech). The ABC subscales have been extensively used in ASD research [65,66]. The irritability subscale (ABC-I) was the primary outcome measure in the pivotal multi-site studies of both risperidone [23,43] and aripiprazole [22,67] in ASD. The ABC-I subscale consists of 16 items and includes assessment of physical aggression, temper tantrums, yelling, screaming, and self-injurious behaviors (see review [68]). The hyperactivity subscale of the ABC (ABC-H) has been used in ADHD-symptom treatment studies in ASD using pharmacological [69], dietary [70], and technology-related interventions [58]. Similarity, the social withdrawal/lethargy subscale (ABC-L/SW) has been used to measure the impact of treatment in ASD-related social impairment [71]. ABC-subscale changes of >25% have been used as a measure of response to treatment in a number of studies, including those focusing on irritability [22,23,43,67] and hyperactivity [43,69,72], and was also used as a measure of response to intervention in this study.

## 3. Methodology

### 3.1 The Technology

The Empowered Brain is an assistive technological tool designed to help improve social communication in children and adults with ASD [34,37].

**Figure 1:**
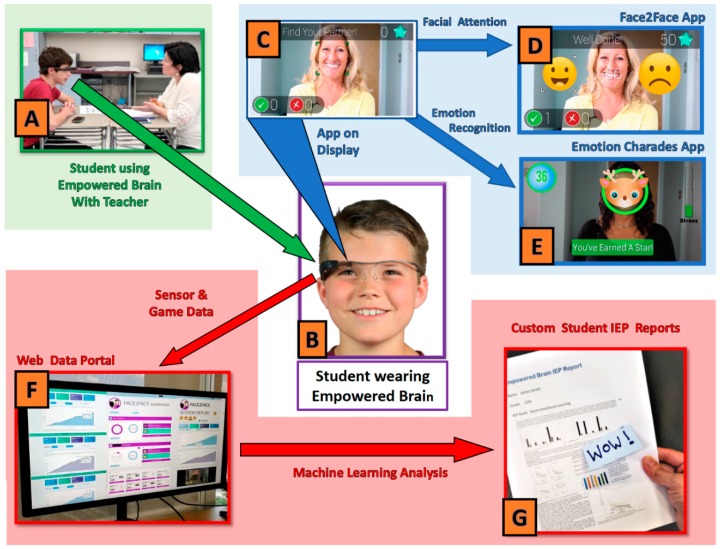
(**A**) Student with Autism Spectrum Disorder (ASD) wears Empowered Brain as he interacts with educator during a 10-min intervention in a typical classroom setting. (**B**) Student looking at display screen of Empowered Brain. (**C**) Screenshot of one of the Empowered Brain apps as would be seen on the optical display of smartglasses. (**D**) Example of the Face2Face app, a game-like experience designed to improve facial attention and mutual eye gaze. (**E**) Example screenshot of Emotion Charades app, a game-like experience that improves emotional understanding through the use of emotional artificial intelligence. (**F**) Example of metrics and qualitative session and student data captured and analyzed by data portal. (**G**) Generation of customized reports for student Individualized Education Programs (IEP) demonstrating learning and skill development.

It is designed to be used for 10-min sessions, during which time the user wears the smartglasses and participates in a semi-structured interaction with a facilitator. The smartglasses provide users with pro-social cues and guidance by visual and auditory feedback delivered through the optical display and speaker. The digital web portal allows students/users and educators to see numerical reports of a user’s in-game performance, measures of social communication, and other metrics, such as attention (Figure 1).

Prior research into the Empowered Brain has provided evidence for its feasibility [54], usability [55], desirability [56], smartglasses sensor accuracy [73], and potential impact on behavioral symptoms [54,58,59,60] (See Table 1). Many individuals with ASD also present with sensory challenges, either hyper- or hypo-reactivity to sensory input [1]. These challenges can lead to extreme responses to tactile sensory, gustatory, and visual stimuli, such as those encountered when hair is combed, teeth are brushed, or glasses are worn. We have previously shown that individuals with ASD can use the Empowered Brain with minimal sensory issues [57]. The physical characteristics of the Empowered Brain make it well suited for use by children with sensory issues engaged in socio-emotional tasks, given that it is lightweight with little nasal bridge pressure, and minimally occludes the user’s face and visual field [56,57].

One of the software modules within the Empowered Brain is Face2Face. When this module is running, the Empowered Brain system detects the extent to which a user is directing his/her attention towards the facilitator. By continuously monitoring metrics of engagement, such as the user’s head positioning relative to the facilitator, the system seeks to motivate and guide the user to remain attentive to the facilitator. The system does this in part by delivering augmented reality cues to the user when they look away from the facilitator. These cues include visually displayed guidance arrows pointing to the facilitator, and cartoon-like masks that are superimposed on the facilitator’s face. These masks attract the user’s attention and motivate him/her to look towards the facilitator. Once the user achieves face-directed gaze, the mask gradually fades and the user is encouraged to maintain gaze with a series of in-game points. The game-like experience is a crucial part of the pro-social element of the system, and improved attention and interaction are rewarded in-game with points, achievements, and unlocked levels.

Session performance data are gathered by the Empowered Brain smartglasses through in-built sensors, which can collect metrics related to the user’s gaze, head movements, blinking, and voice. This information is then sent to the cloud for processing, and subsequently can be reviewed through the Brain Power web portal.

The technology behind the Empowered Brain is based on innovations in software, engineering, and artificial intelligence afforded through relationships with X (formerly Google X, Mountain View, CA, USA), Affectiva (Boston, MA, USA), and Amazon (Seattle, WA, USA).

### 3.2. Study Design

While traditional research methodologies in medicine have emphasized the use of double blind randomized controlled trials for medication-based studies, this approach is much more difficult for studying perception-altering immersive technology in children with neurodevelopmental challenges [74]. In this population, validation of new technologies often relies on feasibility-based studies [74], especially those that incorporate single-case experimental design. Single-case experimental design has been found to be a methodologically robust approach of assessing the outcomes of behavioral interventions [75,76,77,78,79]. An important additional consideration is the impact of technological novelty on this population, where participant motivation to use a technology may decrease after the novelty of the digital approach has been lost. In the case of shorter or single exposure studies, the outcome effect of a particular intervention may be overstated if the effect of novelty, and/or prior use of similar technology, is not taken into consideration [74,80]. Accordingly, in this line of research, we focus on regular (twice-daily) and longitudinal assessment of this intervention.

**Figure 2:**
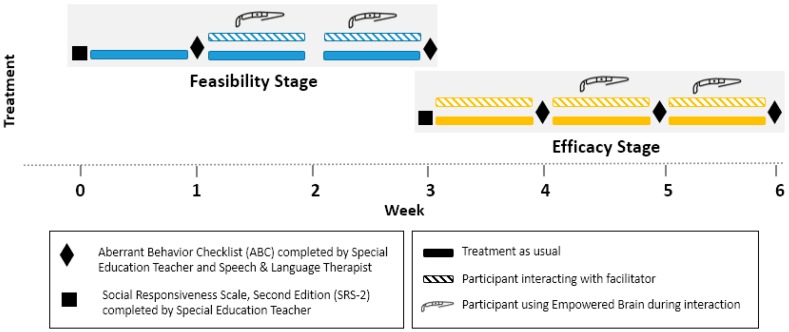
Outline of experimental study methodology across both feasibility and efficacy stages.

The study consisted of two stages over six weeks, and involved four students with ASD and three educators who facilitated the intervention. The first stage assessed the feasibility of the intervention in a school setting over an extended period of time. Following the feasibility stage, the efficacy stage involved a control period, an intervention period, and a subsequent intervention extension period. The total duration of the study was six weeks, with the feasibility stage and controlled efficacy stage each lasting three weeks. Single-case experimental design was used during both stages (Figure 2).

The feasibility stage involved an initial baseline week, where students continued with their regular educational schedule followed by a two-week intervention period. During the intervention period, each student received twice-daily interventions during the Empowered Brain. These intervention sessions lasted 10 min, and were facilitated by an educator (either a special education teacher or an SLT). A baseline ABC was completed by the educators at the end of the baseline and intervention periods. Primary outcomes were changes to the ABC-I, ABC-H, and ABC-L/SW. Additionally, this stage explored the ability of educators to deliver this intervention in a classroom setting concurrently to regular academic instruction.

The efficacy stage of the study followed positive feasibility findings from the first stage. This stage lasted three weeks and involved a one-week control period, a one-week intervention period, and an additional intervention extension period. During the control week, twice-daily 10-min socio-emotional focused conversations were facilitated by the educators without the use of the Empowered Brain system. This was followed by an intervention week where these twice-daily conversations were repeated but with the addition of the Empowered Brain system. Following the intervention week, a one-week intervention extension period was also undertaken to further explore the longer-term effects of the smartglasses augmented intervention.

Additionally, at the end of the study, educators could rate their experience using the system relative to other assistive technologies they have previously used in their educational career. Using a four-point Likert scale, they were able to subjectively rate whether the system was very inferior, inferior, superior, or very superior to other assistive technologies that they had previously used or were currently using.

### 3.3. The Intervention

The intervention consisted of the participant (student) sitting opposite their facilitator (educator) while the participant wore smartglasses running the Face2Face module. The facilitator engaged the participant in a natural conversation regarding relevant academic topics; for example, a project that had been assigned to the student or a recent homework assignment.

The Empowered Brain smartglasses would monitor the attention of the participant to the facilitator’s face, and would provide visual and auditory feedback to the participant when appropriate. As the participant interacted with the facilitator and took heed of the guidance, they would receive in-game points and rewards. The guidance would decrease when the participant demonstrated increased attention to the facilitator, and increase when the participant would look away for a prolonged duration of time. 

The 10-min intervention was provided twice during school days: typically a morning intervention delivered by the special education teacher, and an afternoon intervention delivered by an SLT.

The control week of this study involved twice-daily sessions that were identical to the intervention, with the exception that the Empowered Brain was not worn through the natural conversation between facilitator and participant.

An ABC was completed by the same raters for each participant at the end of the control week, and at the end of the two-week intervention. Each participant had three raters score their behavior: their special education teacher, the SLT, and their caregiver.

### 3.4. The Setting and Participants

The study was conducted in special education and general education classrooms of a public elementary school in Massachusetts. The participants were all students of the school. All participants were required to have a documented diagnosis of ASD and needed to be in receipt of special education under an Individualized Education Program (IEP). Participants were required to have no current or past history of seizures or a seizure disorder.

The participants were recruited as part of a convenience sample. All participants were male with a mean age of 7.5 (range 6.7–8.8 years). The participants had no therapeutic intervention changes during the course of the study, including behavioral therapies or medications. The special education teacher completed a Social Responsiveness Scale, Second Edition (SRS-2) assessment for each participant to confirm impairments in social communication. The SRS-2 is a validated social communication measure used in ASD populations [81]. Post-intervention SRS-2 data are not reported here. Mean participant SRS-2 score was 71 (range 65–82), indicating that all participants have marked impairment in social communication.

### 3.5. The Control and Intervention Facilitators

Three facilitators were involved in this study, all of whom were highly experienced educators, and provided both the control and the intervention sessions. The facilitators included one special education teacher who provided the intervention to all four students, and two SLTs, one of whom provided the intervention for one student, and the other for the remaining three students. Educators were assigned to students that they worked with on a daily basis for their typical school-based treatment.

The special education teacher has a master’s degree in special education, doctoral level experience, and 28 years of early education experience, 11 years of which were in special education. The teacher has educated approximately 30 students with special needs, with the majority of whom having ASD. The teacher typically teaches the same students for 3–4 years. The teacher has experience in assistive technology, and primarily uses iPad tablets with embedded technology or assistive apps.

The first SLT has 28 years of educational practice, with a master’s degree in speech and language pathology. The SLT has taught over 1000 students, of which approximately 150 had/have an ASD diagnosis. The SLT has experience with a range of assistive technology devices including tablets, augmentative communication devices, voice output devices, and text to speech tools.

The second SLT has 20 years of educational experience, a master’s degree, and has taught over 500 students, of whom approximately 25 have had ASD. The SLT has experience with low technology devices, communication boards, and online systems.

### 3.6. Consent and Institutional Review Board (IRB) Status

The use of the Empowered Brain running on multiple head-worn computing devices by children and adults with ASD was approved by Asentral, Inc., IRB, an affiliate of the Commonwealth of Massachusetts Department of Public Health. The study was performed in accordance with relevant guidelines and regulations, and in accordance with the Helsinki Declaration. Written informed consent was obtained from all parents/legal guardians of all minors involved in this study, and written and verbal assent was provided by all participants over the age of 7. Consent to conduct this research was also obtained from all educators involved in the study. Written informed consent was obtained from all parents/legal guardians of all minors involved in this study for the publication of their identifiable information.

## 4. Results

### 4.1. Feasibility Stage

All participants in the feasibility stage were able to complete the two-week intervention period following the baseline week. Their ABC ratings were successfully completed by their special education teacher and their SLT for the baseline week, and at the end of the intervention period (Table 2 and Table 3). The educators who completed the ABC ratings were facilitators of the intervention.

**Figure 3:**
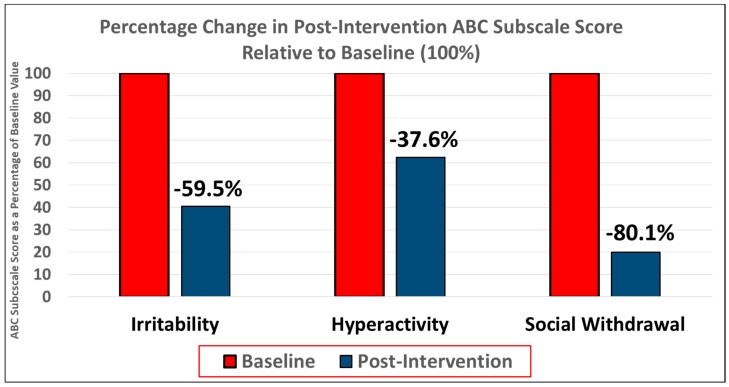
Results of the feasibility stage. Improvement in symptoms of irritability, hyperactivity, and social withdrawal as determined by percentage reduction in ABC subscale scores post-intervention relative to baseline. Irritability was improved by 59.5%, hyperactivity by 37.6%, and social withdrawal by 80.1%.

The students were noted to have had decreased irritability, hyperactivity, and social withdrawal as determined by the reductions in their respective mean ABC subscale scores (Figure 3). The students demonstrated decreased irritability as demonstrated by a 59.5% reduction in mean educator ABC-I post-intervention score compared to baseline. All ABC-I ratings post-intervention were reduced by a minimum of 6 or more points. Additionally, mean student ABC-H scores post-intervention were reduced by 37.6%, with three out of four ABC-H ratings finding a decrease and 1 rating remaining unchanged. Finally, student social withdrawal/lethargy as measured by the mean ABC-L/SW score was reduced by 80.1%.

### 4.2. Controlled Efficacy Stage

All participants in the efficacy stage completed the control, intervention, and intervention extension periods. Their end of control, post-intervention, and post-intervention extension ABC ratings were successfully completed by their special education teacher and their SLTs. The educators who completed the ABC ratings were facilitators of the intervention.

**Figure 4:**
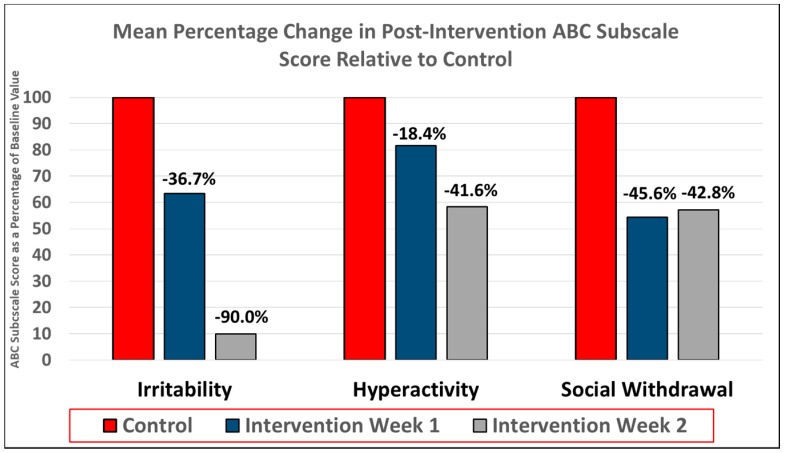
Results of the controlled efficacy stage. Improvement in symptoms of irritability, hyperactivity, and social withdrawal as determined by percentage reduction in ABC subscale scores post-intervention relative to baseline. Irritability, hyperactivity, and social withdrawal were improved at both intervention time points relative to control period.

The participants were noted to have improvement in irritability, hyperactivity, and lethargy following intervention compared to control (Table 4 and Table 5). Additionally, a longer period of intervention, as demonstrated by the two weeks of total intervention by the end extension period, was associated with even greater improvement in irritability and hyperactivity. The mean ABC-I score for the students was reduced by 36.7% at week 1, and by 90% at week 2. It should be noted that these participants had a relatively low ABC-I score at the end of the control period, although three out of four ratings reported student ABC-I score as being zero at the end of the second week of intervention. Hyperactivity was similarly improved following intervention as noted by the reduction in mean ABC-H score of 18.4% at week 1, and 41.6% at week 2. Hyperactivity was improved relative to the control period in all four ratings at intervention week 2, and in three out of four ratings at the end of intervention week 1. Social withdrawal also appeared to be improved following intervention, although, unlike irritability and hyperactivity, the magnitude of the improvement did not increase following the second week of intervention. The ABC-L/SW was reduced by 45.6% at the end of week 1 and 42.8% at the end of week 2 (Figure 4).

### 4.3. Educator perception of Empowered Brain

All three educators who were trained to use Empowered Brain and who facilitated the interview rated the technology as superior (*n* = 2) or vastly superior (*n* = 1) to other assistive technologies that they have previously used.

## 5. Discussion

Technology-based assistive tools are increasingly important tools to address the educational and therapeutic needs of students with ASD. Similarly, these technologies can empower educators and school systems to improve their special education programs and to decrease educator stress and burnout. However, it is important to critically study the feasibility and efficacy of these technology-based interventions. Research into emerging assistive technologies should be conducted in real-world and ecologically valid settings, such as homes, schools, and specialist centers. In this study, we used a multi-stage approach to examine the feasibility and then the efficacy of a longitudinal intervention program using Empowered Brain. Empowered Brain is a smartglasses-based intervention that integrates computerized smartglasses with augmented reality and artificial intelligence to deliver socio-emotional and behavioral skills to students with ASD.

One of the strengths of this study was the use of single-subject experimental design in both the feasibility and efficacy stages; this is important given the robustness of this methodology in assessing outcomes associated with behavioral/psychological interventions [75,76,77,78,79]. The feasibility stage demonstrated that the technology was usable and practical to use concurrent to regular classes, as facilitated by regular school educators. Participants who underwent this feasibility stage demonstrated reduced irritability, hyperactivity, and social withdrawal as rated by educators on a validated scale. The second stage studied the efficacy of the intervention and obtained data from both a control and intervention period. Participants who underwent the efficacy stage demonstrated reduced irritability, hyperactivity, and social withdrawal following the intervention as compared to the control period. Notably, there was an intervention exposure/dose-response relationship linking increased duration of intervention with improvement in irritability and hyperactivity in students. Improvement in social withdrawal appears to have plateaued at the end of the first week of intervention. Our results here are important for two reasons: First, they help to guide the appropriate intervention intensity and duration of our future work involving multisite randomized controlled trials; second, they provide some evidence suggesting that initial interventional novelty did not play a significant role in the improvement seen in these challenges.

Educator feedback on their assessment of the technology was positive, with Empowered Brain rated as being superior or vastly superior to past/current assistive technologies. This is important given that the educators all had considerable experience with assistive technology and 20–28 years of teaching experience.

There are several important limitations to this study. While the use of single-case experimental design was effectively used, studies using a different theoretical framework would help to further validate this technology. Larger-scale studies would allow for more robust statistical interpretation of the results, and potentially greater generalizability. With that said, many of the assistive apps and technologies that are commercially available for the ASD student population have little research validation. There is even less research on digital interventions that combine several fields of emerging technologies, such as smartglasses, augmented reality, and artificial intelligence. Therefore, this research would be viewed as preliminary but promising evidence of the utility of this type of technology. Additionally, this study lasted a total of six weeks. While this is a typical length of time to deploy a technology in an ecologically valid setting, such as a live classroom, we should also bear in mind that behavioral interventions in ASD often last months if not years. Extended longitudinal research could help to study the decaying effects of new technologies. Accordingly, it is recommended that longer duration of intervention be studied as part of future research, a recommendation that is further supported by evidence of a dose/response relationship between increasing duration of intervention and improvement in hyperactivity and irritability.

## 6. Conclusions

The results of this study demonstrate the feasibility and efficacy of Empowered Brain in improving irritability, hyperactivity, and social withdrawal in a sample of students with ASD within a public elementary school setting. This technology was studied as part of a multi-stage process over a six-week period, with both feasibility and controlled efficacy stages of the study using validated single-case experimental design methodology. Further research is required to further elucidate the impact of this technology over a longer time course and with randomized control trial studies.

## Figures and Tables

**Table 1:** Research background and peer-reviewed research findings related to Empowered Brain.

No. of Multisite IRB * Approved Programs	2 (2016 and 2017)
Current Number of Research Sites	8+
Demonstrated Feasibility	Liu et al. [54]
High Usability	Sahin et al. [56] Keshav et al. [59]
High Desirability	Sahin et al. [56]
High Tolerability	Keshav et al. [55]
Safety Study	Sahin et al. [57]
Improvement in Social Communication	Sahin et al. [60] Keshav et al. [59] Liu et al. [54]
Improvement in ADHD **-related symptoms	Vahabzadeh et al. [58] Liu et al. [54]
Demonstration of Positive Teacher Perception	Keshav et al. [59]

* Institutional Review Board; ** Attention Deficit Hyperactivity Disorder

**Table 2:** Feasibility stage ABC subscale scores for Participant 1.

Rater	ABC Subscale	Baseline	Post-Intervention
**Special Education Teacher Rater**	Irritability	21	10
	Lethargy	1	0
	Stereotypy	4	1
	Hyperactivity	11	6
	Inappropriate Speech	0	0
**Speech and Language Therapist Rater**	Irritability	9	3
	Lethargy	7	3
	Stereotypy	0	0
	Hyperactivity	7	7
	Inappropriate Speech	1	0

**Table 3:** Feasibility stage ABC subscale scores for Participant 2.

Rater	ABC Subscale	Baseline	Post-Intervention
**Special Education Teacher Rater**	Irritability	10	0
	Lethargy	11	1
	Stereotypy	7	2
	Hyperactivity	13	4
	Inappropriate Speech	4	4
**Speech and Language Therapist Rater**	Irritability	13	3
	Lethargy	18	5
	Stereotypy	0	1
	Hyperactivity	14	9
	Inappropriate. Speech	2	2

**Table 4:** Efficacy stage ABC subscale scores for Participant 3.

Rater	ABC Subscale	Time Point		
		Control Week	Intervention Week 1	Intervention Week 2
**Special Education Teacher Rater**	Irritability	5	1	2
	Lethargy	2	0	2
	Stereotypy	1	1	1
	Hyperactivity	42	28	20
	Inappropriate Speech	2	2	1
**Speech and Language Therapist Rater**	Irritability	1	1	0
	Lethargy	5	4	2
	Stereotypy	1	0	0
	Hyperactivity	23	14	12
	Inappropriate Speech	1	1	0

**Table 5:** Efficacy stage ABC subscale scores for Participant 4.

Rater	ABC Subscale	Time Point		
		Control Week	Intervention Week 1	Intervention Week 2
**Special Education Teacher Rater**	Irritability	3	1	0
	Lethargy	17	10	9
	Stereotypy	10	5	4
	Hyperactivity	27	19	13
	Inappropriate Speech	3	2	2
**Speech and Language Therapist Rater**	Irritability	2	2	0
	Lethargy	14	11	5
	Stereotypy	1	2	0
	Hyperactivity	7	9	6
	Inappropriate Speech	3	2	1
